# Mind and Health

**Published:** 1882-05

**Authors:** 


### MIND AND HEALTH.

Dr. Kirkbride, of the Pennsylvania Hospital for the Insane, in his report to the Legislature, says, one seventh of the male patients had no regular occupation at the time of attack, while for 1850 the most numerous class were those who had been in some way engaged in agriculture, either as farmers, farmers' wives or daughters. This proportion is not invariable, for in 1862 the same gentleman reports, that of 3947 insane, there were 297 farmers, 170 of their wives, and 95 of their daughters—about one seventh of the whole were from the farm! Of the 4014 patients in the Central Lunatic Asylum of Ohio, as reported to the Legislature for 1862, 1108 were from the farm! The statistics of the insane in Massachusetts show that the largest number of cases were of farmers' wives.

Nor do farmers live the longest. Travelers and natural philosophers average a greater age. The clergyman who devotes his life to study and late hours, who spends three fourths of his existence in-doors, who does not average two hours' daily exercise in twenty-four, who is compelled to an inactivity of body which would seem enough to undermine any constitution, to say nothing of the many depressing influences connected with his office, in listening to the troubled, in counseling the sick, and in waiting upon the dying and the dead, even he often survives the farmer, who rises with the lark to breathe the pure out-door air, whose undisturbed nights, whose supposed independence of the world, whose table is thought to be spread every day with the freshest butter from the dairy and the new-laid eggs, with pure, rich milk from the spring-house, all cool and sweet, vegetables just dug from the ground or pulled from the vine, and melons taken from the garden, berries from the bending bushes, and fruits, luscious, perfect, and ripe, from the orchard within the hour; in short, a class of persons whose whole surroundings are universally believed to be the synonyms of quiet, plenty, and independence, and which would seem to be a full guarantee of a healthful and happy old age; do not attain it as often as some other classes whose habits and modes of life are not, other things being equal, as favorable to longevity.

In an official report made before the Ohio Legislature, in February, 1863, it was stated that the unmarried exceeded the married by seven hundred and sixty. A sufficient reason for this result is found in the fact, that the mental and moral faculties are called into constant activities in married life, while the very multiplicity of its cares, and their changing nature, so diversify the mental operations, that the brain can not dwell long on one subject. The report very significantly adds: "Those whose lives are devoted to physical labor appear to be far more liable to insanity than those whose occupation calls for a larger exertion of the mental faculties."

The want of health, and sanity, and longevity among farmers is not a necessity. Two thousand years ago, when life was less artificial, the oldest, and wisest, and best of men were farmers; their names have come down to us through the long ages intervening, with honor, and they are held in respect in all civilized lands. An able writer in the *Atlantic Monthly* for April, 1863, says: "Xenophon (the most renowned of the world's generals) gained a strength in his Elian fields that carried him into the nineties; Cato lived to be over eighty; Varro wrote his book out by Tusculum at eighty, and survived to counsel with Fundania ten years more. Pliny, too, the Elder, who, if not a farmer, had his country-seats, and left very much to establish our acquaintance with the Roman rural life, was a hale, enduring man, of such soldierly habits and large abstemiousness, as to harvest a good four-score, if he had not fallen under that murderous cloud of ashes from Mount Vesuvius in the year seventy-nine." And who has not read of the good, and great, and wise old Cincinnatus of Roman story?

These examples, with thousands of others in our own day, confirmed by the observation of every intelligent man, prove that a sane mind in a sound body naturally belongs to a farmer's life; and when it is not so, it is because of adventitious circumstances — to wit, a want of intelligence, a want of mental activity on a variety of subjects, and a want also of that intelligent attention to the physical nature, to those laws of health as pertaining to domestic life in relation to personal and habitational cleanliness, to dress, to habits of rest, and sleep, and work, to eating and drinking, and, not least, to a rational cookery, all of which are essential to high health under any circumstances, but more especially so in connection with that artificial mode of life which has so unfortunately extended itself from the city to the town, and village, and hamlet, and to the log-cabin itself.

Not one farmer's wife in fifty is a good bread-baker, and yet bread is the staff of life. The unwise and wicked penuriousness of multitudes of farmers, sends the best and freshest and most luscious articles for the table to market, while he feeds himself, wife, children, and hired help on the milk and butter returned from town, or on the defective fruits and melons of the orchard and the field and garden. There are tens of thousands of them who sit down to their tables for weeks at a time without having a single dish of fresh meat to break the odious sameness of pork and beans, of cabbage and bacon, the hams being sent to market, and spring chickens, and splendid turkeys, and fat young ducks, swarm in the yard, and around the barn, and scatter over the fields—in fact, are to be found everywhere except on their own tables. This is a shame and a disgrace and a sin; because good food, well prepared, is second in importance to nothing that can be named about a farm-house. It is perfectly certain that whatever may be the discomforts, and annoyances, and miseries found in farmers' families, be they more or less, be they physical, mental, moral, social, or domestic, a very large share of them come from bad food, illy prepared, and irrationally eaten.

Let all, especially the young, who look to farming as the future pursuit of life, and who desire to avoid a large share of the ordinary discomforts, privations, unhappiness, and want of health which too often befall so worthy and so large a class of society as farmers' families are, remember these two cardinal suggestions:

First. Never purchase more land for farming purposes than can be paid for without borrowing.

Second. Never attempt to cultivate more than can be thoroughly done with the help which can be readily commanded; for one acre will yield more with a given amount of well-expended labor than two acres will yield with the same.

